# Burnout of Healthcare Workers Based on the Effort-Reward Imbalance Model: A Cross-Sectional Study in China

**DOI:** 10.3389/ijph.2021.599831

**Published:** 2021-02-25

**Authors:** Zhipei Yuan, Dan Yu, Huanyan Zhao, Yanli Wang, Wen Jiang, Dan Chen, Xuan Liu, Xingli Li

**Affiliations:** ^1^ Department of Epidemiology and Health Statistics, Xiangya School of Public Health, Central South University, Changsha, China; ^2^ Hunan Prevention and Treatment Institute for Occupational Diseases, Changsha, China; ^3^ Kailuan Central Hospital, Tangshan, China; ^4^ Tangshan Union Hospital, Tangshan, China

**Keywords:** occupational stress, ERI model, moderating effect, burnout, healthcare workers

## Abstract

**Background:** The effort-reward imbalance (ERI) model is widely used in job stress research. However, few studies using this model have been conducted in developing countries. This study tested the extrinsic and intrinsic hypotheses regarding the burnout of healthcare workers in China with the ERI model.

**Method:** Job stress was assessed by Siegrist’s ERI questionnaire, and burnout was evaluated by the Maslach Burnout Inventory-General Survey (MBI-GS). A total of 1,505 effective respondents were included in the final study. Multiple and hierarchical linear regression was used to analyze the association between components in the ERI model and burnout.

**Results:** Emotional exhaustion and cynicism were positively correlated with ERI and overcommitment. Professional efficacy was positively related to ERI but not to overcommitment. ERI was the determining factor of emotional exhaustion and cynicism. Overcommitment moderated the relationship between ERI and emotional exhaustion and between ERI and cynicism.

**Conclusion:** Changing workplace conditions and increasing personal resilience might alleviate burnout among hospital workers in China. The links between professional efficacy and stressful work environment need further exploration.

## Introduction

Maslach proposed that burnout is a syndrome that includes emotional exhaustion, cynicism, and professional inefficacy [1]. In ICD-11(International Classification of Diseases-11), burnout is described as the result of failed stress management and involves many behaviors and health disorders [2]. Under long-term job stress, the well-being and health of employees can be damaged [3]. Employees who suffer from burnout experience exhaustion of physical resources and energy, indifference to interpersonal relationships, and feel powerless at work. After conducting interviews, observations, and psychometric development work, Maslach came to view burnout as a 3-dimensional construct that included lack of efficacy [4]. Although others have questioned its centrality to burnout, Maslach insisted that lack of efficacy must be included [5]. According to previous reports, the prevalence of burnout among healthcare workers in most regions exceeds 50% [6, 7]. Continuing reform of medical systems in China has increased the demand for quality medical services has soared. Hospital worker teams face challenges such as heavy workloads, patient responsibilities, physical risk, and failure of the system to reward them appropriately [8, 9]. In health care staff, burnout leads to decreased quality of care and even treatment errors [10]. Furthermore, burnout overlaps with anxiety, depression, and other mental disorders [11], with considerable impacts on workers.

The effort-reward imbalance (ERI) model suggests that workers experience job burnout when the effort (e.g., time, energy, and responsibility) they invest does not match the reward (e.g., remuneration, respect, and professional opportunities) they receive [12]. The ERI model summarizes the most significant extrinsic factors of work environments based on reciprocity and equity theories. As an integrative model, it proposes that personal coping strategies also influence stress responses: one such response is called overcommitment. The emotional motives of overcommitment are fear of losing control of their work and a desire for appreciation [13]. Overcommitted workers put unrealistic expectations upon work and invest inappropriate effort. Thus, they are under sustained strain reactions [14]. Indeed, adverse effects might be accentuated when overcommitted workers are in high effort-low reward environments [15]. However, the moderating role of overcommitment in the relationship between stressful work and individual outcomes remains to be explored, even more so in hospital workers.

Previous studies have verified that a lack of reciprocity results in burnout [16]. However, in most of these studies, the components of the ERI model and burnout were not investigated simultaneously. Furthermore, the relative importance of the internal and external components remains controversial [17]. Especially in developing countries, participants were doctors or nurses, the evidence from other healthcare professionals are limited, like pharmaceutical or management staff [18]. Considering the ambiguity of the evidence, we tested the following hypotheses among four different groups (clinicians, nurses, pharmaceutical staff, and management staff) of hospital workers in China: 1. Effort-reward imbalance and overcommitment are both positively associated with every dimension of burnout 2. The relationship between burnout and the effort/reward ratio (ERR) is stronger than the relationship between burnout and effort, reward or overcommitment 3. Overcommitment plays a moderating role in the relationship between reciprocal imbalance and burnout. The results are not only conducive to the development of the ERI model but also provide additional scientific theoretical guidance for the prevention of job burnout.

## Methods

### Design and Participants

**FIGURE 1:**
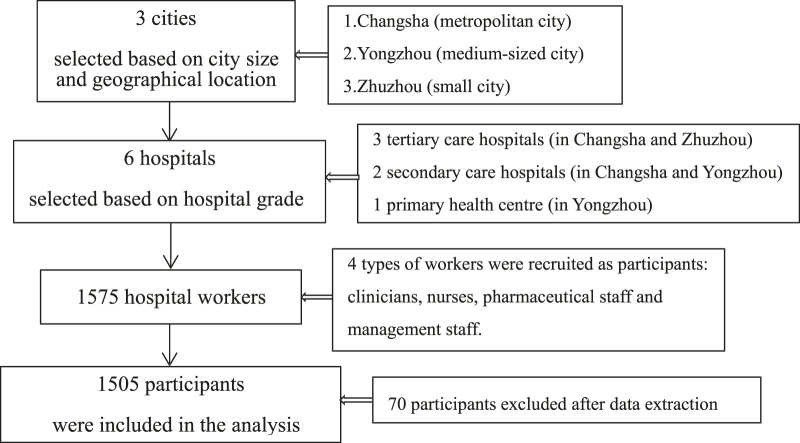
Flowchart of the investigation process.

A cross-sectional study was conducted in Hunan Province from June 2019 to July 2019. Hunan Province, located in central China, has a population of nearly 70 million. Multistage stratified random sampling was performed. Research sites (i.e., hospitals) were selected according to the city size and geographical location. A flowchart of the sampling process was provided in Figure 1. First, three cities were randomly selected: Changsha (metropolitan city), Yongzhou (medium-sized city), and Zhuzhou (small city). Next, three hospitals, two hospitals, and one hospital were randomly selected from Changsha, Yongzhou, and Zhuzhou, respectively. The representative sample included primary health centers, secondary care hospitals, and tertiary care hospitals. Four types of medical workers were recruited as participants: clinicians, nurses, pharmaceutical staff, and management staff. The inclusion criteria were as follows: all of the participants had worked for more than six months; participants had no history of mental illness and had not used psychotropic drugs within one week of participating in the survey, and participants voluntarily participated in the survey and completed the questionnaires. The flowchart briefly described the investigation process (Figure 1).

### Measurements

#### Demographic and Job-Related Factors

Demographic factors included age (<30 years, 30∼ years, ≥40 years), gender (male, female), marital status (single, married/cohabiting, divorced/widowed/separated), and education (junior college and below, undergraduate, graduate and above). Four job characteristics were measured: position, years worked, monthly income, and hours worked per week. The monthly income was divided into <5000 RMB ($710), 5,000∼ RMB, and ≥9000 RMB ($1,260). The four types of positions included clinicians, nurses, pharmaceutical staff, and management/support staff, and the work experience was divided into three groups: less than <5 years, 5∼ years, and ≥10 years. The working hours of medical staff were also considered. The number of hours worked per week was divided into three groups: <40 h, 40∼ h, and ≥70 h.

#### Measurement of Job Stress

Siegrist’s ERI questionnaire was used to measure job stress. The Chinese version of the questionnaire has been characterized with good reliability and validity [19]. The questionnaire consists of 22 items, six of which measure effort (e.g., “in recent years, my work burden is getting heavier and heavier”), 11 measure return (e.g., “in terms of my efforts and achievements, I have the right job prospects”), and five measure overcommitment (e.g., “do you start thinking about work as soon as you get up in the morning?”). All dimensions are scored on a 5-point Likert scale, with higher scores reflecting more effort, reward, and overcommitment. The original score of reward is obtained by reverse scoring. The total score of each dimension is divided by the number of items to obtain the average score. The ratio between effort and reward score is the indicator of ERI. Workers with a ratio >1 (i.e., high effort and low reward) are defined as a high state of tension [20]. The Cronbach α coefficients of the three dimensions of effort, reward, and overcommitment were 0.87, 0.92, and 0.89, respectively.

#### Measurement of Burnout

The Chinese version of the Maslach Burnout Inventory-General Survey was used; this version is characterized by high reliability and validity [21]. There are 16 questions in the Chinese version of the questionnaire, including emotional exhaustion (five items, e.g., “work makes me tired”), cynicism (five items, e.g., “my enthusiasm for work has decreased”), and professional efficacy (six items, e.g., “I am engaged in a valuable job”). The questionnaire is scored by a seven-point Likert scale, with 0 for “never” and six for “every day.” A higher the score in the dimensions of emotional exhaustion and cynicism, and a lower the score in the dimension of professional efficacy, indicated more serious job burnout. The Cronbach α coefficients of the three dimensions of emotional exhaustion, cynicism, and professional efficacy were 0.94, 0.88, and 0.92, respectively.

### Statistical Analysis

IBM SPSS 20.0 software was used for statistical analysis. Unified standards are required when applying a cut-off to determine individuals with or without burnout. Additionally, there are criticisms of the dichotomization of burnout variables. For these reasons, we used continuous scales of burnout in our analysis. Pearson correlation and linear regression were used to assess associations between burnout and the ERI components (hypotheses 1 and 2). Crude and adjusted models (including gender, income, weekly working hours, position, and working years) were tested. The correlation coefficient between burnout and ERI was analyzed through the partial regression coefficient. To test hypothesis 3, we utilized two statistical approaches. In the first approach, we used hierarchical linear regression. Three linear regression equations were established by taking emotional exhaustion, cynicism, and professional efficacy as dependent variables. Demographic characteristics, ERR, overcommitment, and ERR×overcommitment were entered in the equation in order. The statistical significance of the interaction term (ERI × overcommitment) and ΔR^2^ were used to test the interaction hypothesis. In the second statistical approach, the participants were classified into the following four groups using medians of overcommitment and ERR as cut-off points: 1) low ERR, low overcommitment (0-0); 2) high ERR, low overcommitment (1-0); 3) low ERR, high overcommitment (0-1); and 4) high ERR, high overcommitment (1-1). We then used covariance analysis to compare the differences in mean burnout scores between groups. The LSD two-tailed *t*-test was used to perform multiple comparisons.

## Results

Table 1 shows the demographic and work-related characteristics of the 1,505 participants. Of the participants, 1,082 were female (71.89%). The mean age was 34.10 ± 8.87 years, and 43.2% were 30–40 years old. Overall, 68.2% of the participants were married or in cohabitation. More than half (59.7%) had received a college education, and 36.8% had monthly incomes between 5,000 and 9000 RMB (approximately $710–$1,260). Of the participants, 65% worked 40–70 h per week.

**TABLE 1:** Demographic and work characteristics of the participants (n = 1,505).

Variable	*N*	%
Age		
>30	512	34.0
30∼	689	45.8
≥40	304	20.2
Gender		
Male	420	27.9
Female	1,085	72.1
Education		
Junior college or less	348	23.1
Undergraduate	898	59.7
Graduate or above	259	17.2
Marital status		
Single	387	25.7
Married/cohabitation	1,027	68.2
Divorced/widow/separated	91	6.0
Position		
Clinical doctor	359	23.9
Nurse	802	52.2
Pharmaceutical staff	195	13.0
Management or support staff	149	9.9
Years of experience		
<5	337	22.4
5∼	435	28.9
≥10	733	48.7
Monthly income		
<5000 RMB	422	28.0
5,000∼	529	35.1
≥9000 RMB	554	36.8
Weekly working hours		
<40	444	29.5
40∼	911	60.5
≥70	150	10.0

Table 2 shows the mean scores of the burnout subscales and ERI components and the unadjusted linear trend between them. ERR was significantly associated with all three burnout subscales, indicating that high effort, low return, and overcommitment led to exhaustion and cynicism. Interestingly, effort and ERR were positively associated with professional efficacy. We did not find an association between overcommitment and professional efficacy. Of the three dimensions of burnout, emotional exhaustion had the strongest association with ERR (correlation coefficient = 0.593, *p* < 0.01), followed by overcommitment (correlation coefficient = 0.632, *p* < 0.01).

**TABLE 2:** Mean, standard deviation, and correlation between components of ERI and burnout.

Variable	M (SD)	1	2	3	4	5	6
1. Effort	3.24 (0.81)						
2. Reward	3.66 (0.69)	−0.468^**^					
3. ERR	0.96 (0.49)	0.754^**^	−0.792^**^				
4. Overcommitment	2.73 (0.88)	0.679^**^	−0.664^**^	0.708^**^			
5. Emotional exhaustion	2.41 (1.63)	0.570^**^	−0.554^**^	0.593^**^	0.632^**^		
6. Cynicism	1.77 (1.48)	0.411^**^	−0.585^**^	0.534^**^	0.532^**^	0.782^**^	
7. Professional efficacy	4.32 (1.50)	0.194^**^	0.115^**^	0.089^**^	0.039	0.007	−0.074

M, mean; SD, standard deviation.

*p < 0.05, **p < 0.01.

Very similar findings resulted from the analysis of the adjusted models (Table 3). After adjusting for demographic covariates (gender, income, weekly working hours, position, working years), the ERR explained the exhaustion and cynicism of burnout better than overcommitment could (beta = 1.900).

**TABLE 3:** Partial regression coefficient (beta and 95% confidence interval) of three dimensions of burnout for job stress.

Scale	Emotional exhaustion	Cynicism	Professional efficacy
Effort	1.101 (1.015, 1.188)	0.738 (0.652, 0.825)	0.308 (0.215, 0.401)
Reward	−1.277 (−1.377, −1.177)	−1.274 (−1.364, −1.185)	0.273 (0.163, 0.382)
ERR	1.900 (1.762, 2.038)	1.619 (1.485, 1.752)	0.214 (0.058, 0.371)
Overcommitment	1.145 (1.071, 1.219)	0.902 (0.828, 0.976)	0.030 (−0.057, 0.117)

Table 4 displays the results of the hierarchical linear regression analysis. The interaction terms (ERR × overcommitment) accounted for a significant increase of 1.5 and 0.6% in the variance of exhaustion and cynicism, respectively. Therefore, overcommitment moderated the relationship between ERR and exhaustion and between ERR and cynicism.

**TABLE 4:** Hierarchical regression results for variables predicting emotional exhaustion, cynicism, and professional inefficacy with overcommitment as a moderator.

Variable	Emotional exhaustion		Cynicism		Professional inefficacy	
	Β	ΔR^2^	Β	ΔR^2^	Β	ΔR^2^
ERR	1.900^**^	0.304^**^	1.619^**^	0.267^**^	0.214^**^	0.005^**^
Overcommitment	0.791^**^	0.089^**^	0.535^**^	0.049^**^	−0.100	0.002
ERR × overcommitment	−0.407^**^	0.017^**^	−0.239^**^	0.007^**^	0.096	0.001

*p < 0.05, **p < 0.01.

The results (Table 5) indicated that the exhaustion and cynicism scores were significantly higher in hospital workers who reported high overcommitment and high ERR compared with those who reported low overcommitment and low ERR (exhaustion scores: 3.11 ± 0.05 vs. 1.17 ± 0.05, *p* < 0.001; cynicism scores: 2.28 ± 0.05 vs. 1.28 ± 0.05, *p* < 0.001). By contrast, there was no significant difference in the professional efficacy scores between group 1 and 4 (4.41 ± 0.06 vs. 4.27 ± 0.06, *p* = 0.064).

**TABLE 5:** Adjusted^a^ mean levels of burnout scores across the effort-reward ratio (ERR) and overcommitment level combinations.

Groups	*N*	Emotional exhaustion	*p* value^b^	Cynicism	*p value* ^b^	Professional efficacy	*p* value^b^
		M (SE)		M (SE)		M (SE)	
ERR = 0, Overcommitment = 0	606	1.71 (0.05)	Referent	1.28 (0.05)	Referent	4.41 (0.06)	Referent
ERR = 1, Overcommitment = 0	178	2.47 (0.09)	<0.001	1.74 (0.08)	<0.001	4.44 (0.09)	0.738
ERR = 0, Overcommitment = 1	145	2.40 (0.09)	<0.001	1.74 (0.09)	<0.001	4.14 (0.10)	0.014
ERR = 1, Overcommitment = 1	576	3.11 (0.05)	<0.001	2.28 (0.05)	<0.001	4.27 (0.06)	0.064
*p* value		<0.001^c^		<0.001^c^		0.027^c^	

M, mean; SE, standard error.

^a^ Adjusted for gender, income, weekly working hours, position, working years.

^b^ p values obtained from multiple comparisons using LSD test with group 1 (ERR = 0, Overcommitment = 0).

^c^ p values for mean differences obtained from the analysis of covariance (ANCOVA).

## Discussion

In this cross-sectional study, we explored associations between effort-reward imbalance and burnout among hospital workers. Different from other studies that only involved nurses or doctors, here we investigated four types of hospital staff and discussed three dimensions of burnout based on the ERI model. In particular, we found that the ERI model cannot fully explain multidimensional burnout. Our results confirm the hypotheses of the effort-reward imbalance model on emotional exhaustion and cynicism and add to the controversies about the 3-dimensional construct of burnout.

For hypothesis 1, Emotional exhaustion and cynicism were associated with ERI components. Overexertion (increased effort), effort-reward imbalance, and demands of control can trigger exhaustion; thus, workers engage in defensive coping methods (cynicism). Siegrist claimed that a stressful work environment might significantly contribute to psychological distress [22]. Rewards were negatively associated with emotional exhaustion and cynicism and enhance self-efficacy because it can help workers overcoming the difficulties of work [23]. Emotional exhaustion showed a strong positive correlation with ERR, which is consistent with several studies [24, 25]. Exhaustion is the direct response to strain and is identified as the core aspect of burnout. In our study, effort and ERR were positively associated with professional efficacy, which has not been previously found. It shows the positive attitude of medical workers in stressful conditions. A possible reason for this association is that cognitive processing greatly influences the relationship between workload and burnout [26]. Hospital workers with a high level of self-efficacy were prone to consider the strain (e.g., an unequal or excessive workload) to be a positive challenge, not a hindrance [27]. However, we did not find an association between overcommitment and professional efficacy. As a positive dimension of burnout, professional efficacy seems to be a vital element of engagement. However, setting unrealistic goals does not appear to motivate engagement.

Regarding hypothesis 2, compared with other the single components of the ERI model, ERR was a stronger predictor of emotional exhaustion and cynicism. The results remained consistent after adjusting for covariates (see Table 3), similar to previous studies [28]. As an external combined measure, the ERR could aptly represent the features of work stress in the current Chinese medical environment when compares with the psychological risk factor (overcommitment) [29]. Thus, organizational intervention should be the basis of reform for long-term benefits. Effective solutions include providing positive feedback (such as higher compensation or more opportunities for promotion) that matches the input, decreasing working hours, and providing psychosocial intervention training. Burnout prevention teams (BPTs) [29] can help organizations assess psychological stressors and physical workplace factors. Individual-focused strategies such as mindful stress reduction and cognitive behavioral therapy can help to increase personal resilience; this can improve one’s negative state, aid in rebound from emotional distress, and help workers maintain energy in their position [30]. However, it would be meaningful to assess whether these practices to maintain performance can themselves become a source of burnout. Further studies are needed to explore the weight of internal and external components and the way in which they combine and then discuss how individual and organizational interventions combine to effectively improve the well-being of hospital workers.

Compared to the other components of the ERI model, effort had a closer relationship with professional efficacy. As professionals dedicated to serve and help patients, Chinese hospital workers invested more extrinsic effort into maintaining a high level of professional efficacy, which seems to be a positive coping mechanism in the short-term but may lead to energy depletion and disengagement over time. A previous study showed that individuals who continued to feel exhaustion and cynicism eventually experienced professional inefficacy [31].

Our results partially support hypothesis 3. Overcommitment synergistically impacted the ERI to exacerbate exhaustion and cynicism, but such interaction did not affect efficacy; these findings were in accordance with previous studies [32, 33]. When reciprocity failed, overcommitted workers were more vulnerable to exhibit passive performance. The relationship between professional efficacy and the ERI model deserves further investigation. Previous studies have found that efficacy is weakly related to job stressors but is strongly related to job resources [24]. Some scholars have found [34] that professional efficacy is independent of exhaustion and cynicism.

## Limitations

There were several limitations to this study. First, it was a cross-sectional study, so our findings cannot verify a causal relationship among these variables. Second, the data were acquired through participant self-reporting; as such, information bias cannot be avoided. Workers with burnout are more likely to express negative emotions. The association between burnout and the ERI could have been overestimated. The stress response is a dynamic and complicated process, and the current design could not estimate a lag effect. Third, the ERI model could not fully explain professional efficacy. We cannot exclude the limitations of the ERI hypotheses and the bias caused by cross-sectional studies and self-reported data. Future studies should incorporate a more rigorous study design and take into account more variables, such as lifestyle factors, work satisfaction, and disease conditions. Despite the above limitations, this is one of the few studies documenting the role of stressful work in burnout among four different groups of hospital workers in China, a rapidly developing country. Moreover, it tests the moderation hypothesis of the ERI model, which has rarely been done. This research will be helpful in future studies of the extrinsic and intrinsic hypotheses of the ERI model and in the development of burnout interventions for hospital workers.

## Conclusion

Our findings partially supported the extrinsic and intrinsic hypotheses of the ERI model. Compared with personal factors, unfavourable work conditions were more prominent determinants of burnout in the health care sector. Burnout interventions should primarily be based on workplace changes but also on increasing personal resilience. Considering the weak correlation between professional efficacy and the components of the ERI model, the links between job burnout and stress-coping require further exploration. In future studies, we aim to integrate several models or combine quantitative and qualitative methods to investigate work-related health problems.

## Data Availability

The datasets presented in this article are not readily available because they are part of an ongoing study. Requests to access the datasets should be directed to yyyuan@163.com.

## Abbreviations

ERI, effort-reward imbalance; ICD-11, international classification of diseases-11; MBI-GS, maslach burnout inventory-general survey.
